# Risk factors for the in-hospital mortality of CRRT-therapy patients with cardiac surgery-associated AKI: a single-center clinical study in China

**DOI:** 10.1007/s10157-022-02274-1

**Published:** 2022-09-09

**Authors:** Yun Jiang, Jianle Chen, Yamin Yu, Fan Yang, Mohsin Hamza, Ping Zou, Ailing Wen, Huihui Wu, Yide Zhang

**Affiliations:** 1 Cardiothoracic Department, The Affiliated Hospital of Nantong University, Jiangsu, China; 2 Nehrology Department, The Ningxiang People’s Hospital, Hunan, China; 3 National Clinical Research Center of Kidney Diseases, Jinling Hospital, Nanjing University School of Medicine, Jiangsu, China; 4 Nephrology Department, The Affiliated Hospital of Nantong University, Jiangsu, China; 5 Research Center of Clinical Medicine and Nephrology Department, The Affiliated Hospital of Nantong University, Jiangsu, China

**Keywords:** CS-AKI, CRRT, In-hospital death, Risk factors

## Abstract

**Objective:**

We retrospectively analyzed risk factors on in-hospital mortality in CRRT-therapy patients with open cardiac surgery (CS)-induced acute kidney injury (AKI), to provide the clinical basis for predicting and lowering the in-hospital mortality after CS.

**Methods:**

84 CS-AKI patients with CRRT were divided into survival and death groups according to discharge status, and the perioperative data were analyzed with R version 4.0.2.

**Results:**

There were significant differences between the two groups, including: urea nitrogen, Sequential Organ Failure Assessment (SOFA) score and vasoactive-inotropic score (VIS) on the first day after operation; VIS just before CRRT; SOFA score and negative balance of blood volume 24 h after CRRT; the incidence rate of bleeding, severe infection and MODS after operation; and the interval between AKI and CRRT. Univariate logistic regression analysis showed that SOFA score and VIS on the first day after operation; VIS just before CRRT; VIS and negative balance of blood volume 24 h after CRRT; the incidence rate of bleeding, infection and multiple organ dysfunction syndrome (MODS) after operation; bootstrap resampling analysis showed that SOFA score and VIS 24 h after CRRT, as well as the incidence of bleeding after operation were the independent risk factors.

**Conclusion:**

Maintaining stable hemodynamics and active prevention of bleeding are expected to decrease the in-hospital mortality.

**Supplementary Information:**

The online version contains supplementary material available at 10.1007/s10157-022-02274-1.

## Introduction

In recent years, tremendous progress has been made in the field of cardiac surgery. Therefore, the incidence rate of CS-AKI is also on the rise, which not only affects the surgical efficacy and prolongs the ICU stay time, but also increases the in-hospital mortality rate. At present, although CRRT has been recognized as an optimal and effective treatment for CS-AKI [1], but the mortality rate still remains as high as 50% [2].

## Objects and methods

### Objects

84 patients in our hospital from January 2013 to August 2021 in cardiothoracic intensive care unit were enrolled. All patients wrote informed consent, and all protocols were approved by our hospital’s institutional review board (IRB number: 2020-K046). Every participant agreed that personal medical information would be legally and reasonably used. Moreover, their privacy will be fully protected. The enrollment criteria included: (1) age over 18 years; (2) coagulation function and other indicators within the normal range; and (3) AKI patients who received CRRT after cardiac surgery. AKI was defined according to the KDIGO criteria [3]: increase in serum creatinine ≥ 0.3 mg/dl within 48 h; or increase in serum creatinine ≥ 1.5 times baseline; or urine volume < 0.5 ml/kg/h for 6 h. The baseline of sCr was defined as the lowest value monitored during hospitalization and follow-up. The exclusion criteria were as follows: (1) pre-existing chronic renal insufficiency; (2) CRRT less than 24 h. CRRT indications included: urine volume and/or serum creatinine values meeting the criteria for AKI diagnosis after cardiac surgery [4], and deterioration of the patient's condition with sustained oliguria, severe hyperkalemia and acidosis even after intensive intervention, such as diuretics, volume expansion and vasoactive agents. All 84 patients in the study were divided into a survival group and death group according to the hospital discharge status.

### Methods

Patient´s data were collected: (1) preoperative data: gender, age, body mass index, history of hypertension and diabetes, preoperative LVEF, cardiac function assessment according to NYHA; (2) intraoperative data: extracorporeal circulation time, aortic occlusion time, blood transfusion, lactic acid peak, blood glucose peak; (3) postoperative data: lab test reports for routine blood tests, liver and kidney function, lactic acid level, potassium, SOFA score and VIS [5], which were collected on the first day after surgery, before CRRT and 24 h after CRRT, as well as the state of volume after 24 h of CRRT. Additionally, postoperative hypoxemia, bleeding, infection, low cardiac output syndrome (LCOS), and secondary tracheal intubation, the duration of mechanical ventilation (MV), the interval time between operation and AKI, as well as the interval time from AKI to CRRT were collected in detail. PaO_2_ < 60 mmHg was diagnosed as hypoxemia; the bleeding specifically referred to pulmonary, intracranial or gastrointestinal hemorrhage after operation, or necessity for an additional operation to control bleeding after the end of the sternotomy; infection was defined as a new surgical-site infection, positive blood culture, or development of postoperative pneumonia.

### Statistical methods

Data were analyzed with R version 4.0.2 (Free Software Foundation, Boston, Massachusetts), Shapiro–Wilk tests were conducted to test the distribution of continuous variables. Normally distributed variables were represented as mean ± standard deviation, and skewed distributed variables were represented as median (inter quantile range, IQR). Paired *t* tests or Wilcoxon signed-rank tests were applied to compare the differences of paired continuous variables. Independent continuous variables were analyzed by Student’s *t* test or Mann–Whitney *U* test. Categorical variables were represented as the frequency (percentage) and were analyzed by the chi-square test or fisher exact test. Variables with clinical significance were chosen for univariate logistic analysis. The statistically significant variables (*P* < 0.05) in the univariate logistic analysis were included in the multivariate logistic regression analysis to identify the independent risk factors. The variables mentioned above were further verified by bootstrap resampling analysis. Two-tailed *P* < 0.05 indicated a statistically significant difference.

## Results

57 were males and 27 females, with an average age of 61.00 ± 13.35 years. There were 60 patients in the survival group, and 24 in the death group; the in-hospital mortality rate was 28.57%. The preoperative data analysis showed there were no statistically significant differences between survival and death group (Table 1).

**Table 1:** Preoperative general information in the survival and death groups

General information	Survival group (60 cases)	Death group (24 cases)	*P*
Gender (cases, male %)	41 (68)	16 (67)	1
Age (year)	62.5 (53.5, 73)	63 (54.75, 73.25)	0.988
Body weight index (kg/m^2^)	24.74 (23.28, 25.5)	24.6 (22.67, 26.1)	0.961
Hypertension (cases %)	27 (55)	11 (54)	1
Diabetes (cases %)	13 (22)	7 (29)	0.656
LVEF, Median (Q1,Q3)	0.58 (0.56, 0.64)	0.56 (0.5, 0.62)	0.098
Class III-IV of NYHA (cases)	11 (18)	9 (38)	0.114
Moderate and severe pulmonary hypertension (cases %)	31 (52)	13 (54)	1

The intraoperative data analysis including the duration of extracorporeal circulation and aortic occlusion, the blood transfusion volume, intraoperative lactate peak, as well as the incidence rate of patients with a blood glucose peak > 11.1 mmol/L showed no statistically significant differences between two groups (Table 2).

**Table 2:** Intraoperative data in the survival and death groups

Data	Survival group (60 cases)	Death group (24 cases)	*P*
Duration of extracorporeal circulation (min)	172 (135.75, 229.75)	159 (134.5, 183)	0.331
Aortic occlusion time (min)	109 (82.5, 131.75)	89.5 (79.25, 108.75)	0.17
Blood transfusion (ml)	400 (200, 900)	600 (187.5, 800)	0.676
Lactic acid peak (mmol/L)	4.1 (3.13, 5.82)	5.9 (3.65, 7.4)	0.062
Blood sugar peak > 11.1 mmol/L (cases %)	39 (65)	15 (62)	1

Statistical analysis showed significant differences between the two groups, including the urea nitrogen, SOFA score and VIS on the first day after operation; VIS just before CRRT, SOFA score and negative balance of blood volume 24 h after CRRT, the incidence rate of bleeding, severe infection and MODS after operation time of MV and the interval from AKI to CRRT (Table 3).

**Table 3:** Postoperative variables in the survival and death groups

Data	Survival group (60 cases)	Death group (24 cases)	*P*
First day after surgery			
WBC (× 10^9^/L)	10.95 (9.1, 13.55)	12.6 (10.6, 14.2)	0.217
Platelets (× 10^9^/L)	100.5 (68, 139)	96 (52, 151.5)	0.744
Total bilirubin (μmol/L)	24.6 (19.8, 35.02)	25.45 (14.88, 35.22)	0.976
Creatinine (μmol/L)	152 (122.75, 197.75)	172 (110, 228.75)	0.847
Urea nitrogen (mmol/L)	11.6 (10.3, 14.22)	8.2 (7.78, 12.65)	0.025*
Lactate peak (mmol/L)	3.1 (2.28, 4.18)	4.1 (2.93, 5.43)	0.076
SOFA score	12 (12, 17.75)	17 (15, 21)	0.008*
VIS	8.25 (5, 13.12)	16 (8.5, 24)	0.001*
Pre-CRRT			
WBC (× 10^9^/L)	12.65 (10.47, 16.6)	11 (8.97, 15.43)	0.155
Platelets (× 10^9^/L)	93.5 (60, 160.75)	94.5 (69.25, 128.5)	0.894
Total bilirubin (μmol/L)	22.1 (15.62, 35.6)	23.6 (16.58, 34.5)	0.736
Creatinine (μmol/L)	251.99 ± 95.05	227 ± 90.39	0.265
Urea nitrogen (mmol/L)	22 (16.3, 29.63)	17.8 (10.92, 28.18)	0.109
Lactic acid (mmol/L)	3.35 (2.2, 4.3)	3.65 (2.48, 4.95)	0.186
SOFA score	14 (13, 15)	17 (15.5, 19.5)	0.054
VIS	14.25 (10, 20.62)	28 (23.5, 40.12)	< 0.001*
K^+^(mmol/L)	4.4 (4.18, 4.7)	4.3 (4.15, 4.8)	0.933
24 h post CRRT			
WBC (× 10^9^/L)	13.1 (10.55, 16.55)	13.05 (8.4, 17.18)	0.699
Platelets (× 10^9^/L)	106 (65, 141.25)	77 (51, 126)	0.434
Total bilirubin (μmol/L)	24.4 (19.92, 31.3)	20.75 (15.7, 27.65)	0.216
Creatinine (μmol/L)	150 (107.5, 194)	124 (104.5, 160.25)	0.198
Urea nitrogen (mmol/L)	13.9 (10.3, 17.95)	11.5 (8.47, 16.78)	0.102
Lactic acid (mmol/L)	1.5 (1.37, 2.2)	1.85 (1.5, 2.38)	0.076
SOFA score	12 (12, 12.75)	18 (15, 18.5)	< 0.001*
VIS	9.25 (7, 13)	29 (15.75, 50.25)	< 0.001*
Negative balance of blood volume (cases %)	47 (78)	9 (38)	< 0.001*
Other information			
Hypoxemia (cases %)	30 (50)	18 (75)	0.065
Bleeding (cases %)	8 (13)	13 (54)	< 0.001*
Infection (cases %)	15 (25)	18 (75)	0.001*
LCOS (cases %)	26 (43)	16 (67)	0.091
MODS (cases %)	21 (35)	21 (88)	< 0.001*
Twice tracheal intubation (cases %)	16 (27)	12 (50)	0.073
Duration MV (min)	123 (68.12, 228.94)	206.12 (106.67, 404.95)	0.044*
Interval time from surgery to AKI (h)	49.1 (23.4, 74)	79.45 (33.13, 132.8)	0.147
Interval time from surgery to CRRT (h)	55.15 (32.05, 86.4)	80.68 (34.22, 145)	0.105
Interval time from AKI to CRRT (h)	5.75 (4.25, 8.14)	8.1 (6.21, 10.3)	0.035*

*There is a statistically significant difference

The data including SOFA score and VIS, VIS just before CRRT, VIS and negative balance of blood volume 24 h after CRRT, the incidence rate of bleeding, infection and MODS after operation and duration of MV analyzed by univariate logistic regression analysis showed statistical significance; however, multivariate logistic regression analysis showed that only VIS 24 h after CRRT and the incidence rate of bleeding after operation were independent risk factors for in-hospital mortality in CS-AKI patients with CRRT (Table 4). Bootstrap resampling analysis further verified that SOFA score 24 h after CRRT was also the independent one (appendix, Supplement Table 1).

**Table 4:** The results of univariate and multivariate logistic regression analysis

Data	Univariate analysis OR (95% CI)	*P*	Multivariate analysis OR (95% CI)	*P*
Urea nitrogen (First day after surgery)	0.934 (0.838,1.042)	0.221		
SOFA score (First day after surgery)	1.208 (1.024,1.426)	0.025*		
VIS(First day after surgery)	1.081 (1.024, 1.140)	0.005*		
VIS (Pre-CRRT)	1.096 (1.046, 1.148)	< 0.001*		
SOFA score (24 h after CRRT)	1.919 (1.310, 2.810)	0.001*	1.321 (1.001–1.744)	0.05
VIS (24 h after CRRT)	1.138 (1.068, 1.212)	< 0.001*	1.096 (1.024, 1.173)	0.008*
Negative balance of blood volume (24 h after CRRT)	0.166 (0.059, 0.465)	0.001*	0.326 (0.059, 1.787)	0.326
Bleeding	7.682 (2.570, 22.960)	< 0.001*	10.826 (1.824, 64.257)	0.009*
Infection	9.000 (3.016, 26.855)	< 0.001*	3.120 (0.581, 16.754)	0.184
MODS	13 (3.470, 48.709)	< 0.001*	2.200 (0.319, 15.193)	0.424
Duration of MV	1.002 (1.000, 1.004)	0.011*	1.001 (0.999, 1.004)	0.298
Interval time from AKI to CRRT	1.031 (0.944, 1.125)	0.05		

*There is a statistically significant difference

## Discussion

Cardiorenal syndrome is a clinical vicious cycle that involves both heart and renal dysfunction. This syndrome has high morbidity and mortality [6, 7]. CS-AKI is usually caused independently or interactively by multiple factors, such as inflammation, ischemia and nephrotoxicity. After CS-AKI, the increased volume of systemic circulation and cardiac preload may lead to heart failure, ultimately gives rise to hemodynamic disorder aggravation and perpetuation of this vicious cycle. CRRT is an effective solution which can not only remove inflammatory mediators and metabolic toxins, but also promote hemodynamic stability and maintain the balance of fluid, electrolytes and acids/bases. Many previous researches showed that CRRT was associated with better in-hospital and long-term survival of patients developing AKI after cardiac surgery [8–10]. However, the in-hospital mortality rate remains high, Perez-Valdivieso [11] reported 50% of in-hospital mortality rate, Han [12] reported 60% of mortality rate within 90 days after surgery, our research showed 28.57% of in-hospital mortality rate. It is a big challenge for cardiac surgeons and nephrologists, the potential risk factors are urgently needed to uncover. Many studies have been done and attempted to figure out potential risk factors for the high mortality, including intraoperative transfusion, high lactic acid, low mean arterial pressure, timing of CRRT, positive blood volume post CRRT, duration of MV and post-operation bleeding, different authors held different opinion according to their own studies [13–20].

Our study showed that the urea nitrogen, SOFA score and VIS on the first day after operation, VIS just before CRRT, SOFA score, VIS and negative balance of blood volume 24 h after CRRT, the incidence rate of bleeding, severe infection and MODS after operation, duration of MV, as well as the interval from AKI to CRRT were significantly different in two groups, all the variates were analyzed with univariate logistic regression, we found that the SOFA score and VIS on the first day after operation, VIS just before CRRT, VIS and negative balance of blood volume 24 h after CRRT, complicated bleeding, infection and MODS after operation, as well as duration of MV might impact the in-hospital mortality rate. Because of small sample, we selected VIS and negative balance of blood volume 24 h after CRRT, bleeding, infection and MODS after operation and duration of MV for multivariate logistic regression analysis, only VIS 24 h after CRRT and complicated bleeding were showed as the independent risk factors. The SOFA score was originally developed as a severity score for sepsis [21]. However, it may be affected by many confounding factors [22]. Pistolesi [23] showed that the SOFA score after CRRT was negatively related with in-hospital mortality. In this study, the SOFA score after CRRT was significantly different between the two groups, the univariate logistic regression analysis did show statistical significance, bootstrap resampling approach further supported the result above, which was consistent with previous studies.

The VIS was originally introduced by Gaies et al. based on Wernovsky formula [5]. Yamazaki [24] showed that VIS could accurately predict mortality after heart surgery. This study coincidently showed that the death group had a significantly higher VIS on the first day after operation, before CRRT and 24 h after CRRT, which is in agreement with a previous report by Yamazaki [24]. Vasoactive drugs can improve cardiac output and contractility, mitigate hypotension, and enhance perfusion of important organs, but these drugs also cause to higher cardiac oxygen consumption and blood redistribution, which can lead to insufficient blood supply to other organs, further exacerbating metabolic acidosis, myocardial ischemic necrosis, liver and kidney dysfunction, and peripheral circulation failure. In this regard, a higher VIS reflecting more severe hemodynamic disorder can be a reasonable clinical marker for assessing the cardiovascular condition after cardiac surgery [25]. CRRT can remove cytokines and prominently lower the dose of vasoactive drugs [26], tapering vasoactive drugs may improve microcirculation, allow aerobic metabolism to be acquired more easily and decrease the production of inflammatory mediators accordingly, and further improve organ function. This study showed that the VIS 24 h after CRRT was an independent risk factor.

The complication of bleeding was reported as an important marker of treatment safety during CRRT [27]. According to this study, complicated bleeding is an independent risk factor for in-hospital death in these patients. The critically complicated events including pulmonary, gastrointestinal, intracranial, or hepatic hemorrhage could lead to relevant organ dysfunction, even trigger MODS and DIC, which undoubtedly contributed to higher mortality. In addition, increased thoracic drainage and bleeding of the gastrointestinal tract and airway can give rise to down titrated anticoagulant doses, or even discontinuation of anticoagulants during CRRT, which can lead to a potential blockage of extracorporeal circulation pipelines and CRRT inefficiency. This result also indicated that effective management and prevention of bleeding can improve the prognosis of these patients.

Previous studies have indicated that hypervolemia increases the mortality rate in patients with CS-AKI [16, 17], and a negative fluid load balance within 3 consecutive days after CRRT may decrease the mortality rate [28]. In this study, the percentage of cases with a negative fluid balance after 24 h of CRRT was significantly different between the two groups (*P* < 0.001), which also showed statistical significance by univariate logistic regression analysis (OR = 0.166, *P* = 0.001), but no statistical significance by multivariate logistic regression analysis (*P* = 0.148). It is possible that 24 h of CRRT may be too short to show significant differences in fluid homeostasis. Additionally, the small number of clinical observations may impact the significance of the statistical results.

There are still no clear guidelines for CRRT in CS-AKI, the optimal time point for the initiation of CRRT remains to be elucidated. Han [12] showed that the timely and effective development of CRRT will significantly reduce the mortality of CS-AKI patients. After AKI occurs following cardiac surgery, if timely intervention is not available, the condition will further deteriorate, and it will be difficult to achieve satisfactory treatment results in the later stage. However, some studies showed that early CRRT strategy was not associated with a lower risk of death in these patients [10, 29]. In this study, there was statistical significance between the survival group and the death group (*P* = 0.035). However, the interval time from AKI to CRRT was not related to in-hospital mortality by univariate logistic regression analysis (*p* = 0.05). Recently, some scholars have suggested that the indications for CRRT should be changed from narrowly defined renal replacement to systemic organ support, and therapeutic principle should be shifted from a conventional remedy for life-threatening conditions to early individualized requirements for fluid management, systemic inflammation elimination and metabolic toxin clearance [30–32]. Finally, results suggest that the AKI to CRRT interval is not an independent risk factor for the in-hospital mortality of CS-AKI patients.

In conclusion, the SOFA score and VIS 24 h after CRRT, as well as the incidence rate of complicated bleeding after operation are independent risk factors for in-hospital mortality in CS-AKI patients with CRRT, timely treatment of maintaining stable hemodynamics and active prevention and treatment of bleeding complication are expected to decrease the in-hospital mortality, and improve the prognosis of these patients.

### Limitation

This study has several limitations. First, our sample size is relatively small, it is only a single-center study, larger-scale and multicenter clinical observational studies on CS-AKI patients with CRRT are needed to clarify the risk factors for in-hospital mortality. Second, we did not follow up these patients after discharge.

## Supplementary Information

Below is the link to the electronic supplementary material.

Supplementary file1 (DOCX 15 kb)
