# Supplementary material for: Risk factors for the in-hospital mortality of CRRT-therapy patients with cardiac surgery-associated AKI: a single-center clinical study in China
Source: Clin Exp Nephrol. 2022 Sep 9;26(12):1233–9. doi: 10.1007/s10157-022-02274-1 (PMC9668795; doi:10.1007/s10157-022-02274-1)
Supplement: Supplementary file 1 — Supplementary file1 (DOCX 15 kb) [file 10157_2022_2274_MOESM1_ESM.docx]

supplemental table 1: results of bootstrap resampling analysis

|  | *Coeff* | Bias | Std.Err. | 95% CI | | *P* |
| --- | --- | --- | --- | --- | --- | --- |
| SOFA score (24h after CRRT) | 0.278 | 7.129 | 163.960 | -0.79 | 14.401 | 0.028* |
| VIS (24h after CRRT) | 0.092 | 2.137 | 39.872 | 0.03 | 7.827 | 0.002* |
| Negative balance of blood volume (24h after CRRT) | -1.122 | -18.339 | 523.954 | -58.731 | 3.634 | 0.198 |
| Bleeding | 2.382 | 78.969 | 1808.495 | -0.554 | 144.102 | 0.007* |
| Infection | 1.138 | 8.655 | 76.458 | -2.491 | 103.109 | 0.227 |
| MODS | 0.789 | 6.849 | 661.646 | -17.879 | 23.897 | 0.332 |
| Duration of MV | 0.001 | 0.32 | 0.702 | -0.01 | 0.067 | 0.227 |
